# A modified CO-related EIT parameter was used to evaluate pulmonary ventilation-perfusion ratio during prone position and inhaled nitric oxide therapy: a case report

**DOI:** 10.3389/fmed.2025.1598248

**Published:** 2025-05-26

**Authors:** Jing Xu, Ming Zhong, Di Liu, Jiayi Guan, Xiaoling Qi, Ruoming Tan, Pengcheng Li, Zhanqi Zhao, Hongping Qu, Jialin Liu

**Affiliations:** ^1^Department of Geriatrics, Ruijin Hospital, Shanghai Jiao Tong University School of Medicine, Shanghai, China; ^2^Department of Critical Care Medicine, Ruijin Hospital, Shanghai Jiao Tong University School of Medicine, Shanghai, China; ^3^Department of Industrial Engineering, Tsinghua University, Beijing, China; ^4^School of Biomedical Engineering, Guangzhou Medical University, Guangzhou, China

**Keywords:** acute respiratory distress syndrome, electrical impedance tomography, inhaled nitric oxide therapy, V/Q ratio, case report

## Abstract

**Introduction:**

Assessment of the V/Q ratio is crucial for understanding the pathophysiology of iNO therapy and prone position in ARDS patients. Recently, the concept of the absolute V/Q ratio measured by EIT has emerged. In this study, we first describe a case where a modified EIT parameter was employed to clinically monitor the absolute V/Q ratio in an ARDS patient during both prone positioning and iNO therapy.

**Case presentation:**

This report describes the case of a 69-year-old ARDS patient with refractory hypoxemia who underwent prone position and iNO therapy. The patient exhibited a positive response to the treatment, showing improved oxygenation and absolute V/Q. A modified EIT-derived parameter, the cardiac output (CO)-related V/Q match index, was utilized to evaluate the absolute V/Q ratio, demonstrating improved consistency with the oxygenation index compared to conventional indicators.

**Conclusion:**

This case elucidates the significance of the EIT-derived parameter—CO-related V/Q match index, revealing its benefits in evaluating the V/Q ratio under the various treatment strategies when compared to traditional ones.

## Introduction

The ventilation/perfusion (V/Q) ratio plays a crucial role in assessing acute respiratory distress syndrome (ARDS) severity and guiding treatment decisions (1). While electrical impedance tomography (EIT) offers a non-invasive way to monitor lung ventilation and perfusion at the bedside (2), accurately evaluating the absolute V/Q ratio for the entire lung remains challenging. This difficulty arises because conventional EIT-based assessments of V/Q matching do not account for the impact of cardiac output (3), a key determinant in the matching of blood flow to ventilated lung areas. A recent study sought to overcome this shortcoming by incorporating cardiac output metrics, thereby facilitating the computation of absolute V/Q ratios in different lung regions (3). Despite these advances, the study did not succeed in delineating a definitive parameter that encapsulates the comprehensive lung V/Q match. Consequently, we devised an innovative parameter derived from EIT, termed the cardiac output (CO)-related V/Q match index. This parameter was derived by aggregating the variance in the absolute V/Q ratio at each pixel, as detected by EIT, in relation to the optimal V/Q value.

In this report, we present the case of a 69-year-old patient with ARDS who underwent thoracoscopic radical surgery. Postoperatively, the patient encountered severe hypoxemia, and EIT assessment indicated a significantly impaired V/Q ratio. To enhance oxygenation, the patient was treated with prone positioning and inhaled nitric oxide (iNO) therapy. Throughout the treatment, the conventional V/Q ratio assessed via EIT did not reliably indicate the trend of changes in the patient’s oxygenation status. In contrast, CO-related V/Q match index showed a robust correlation with the patient’s oxygenation fluctuations. The modified index provided a nuanced understanding that partially elucidates the physiological mechanisms at play in the efficacy of prone positioning and inhaled nitric oxide (iNO) therapy for the management of ARDS. The deployment of this modified parameter has yielded encouraging outcomes, delivering valuable insights that can inform and guide clinical treatment strategies for ARDS patients.

## Case presentation

### Patient history

A 69-year-old male patient who underwent thoracoscopic radical surgery for esophageal cancer developed acute respiratory distress syndrome (ARDS). Pulmonary *Klebsiella pneumoniae* infection was identified based on the sputum culture results. Due to worsening oxygenation and infection progression, he was transferred to the intensive care unit (ICU). Tracheostomy were initiated two days before the admission. Past medical history includes hypertension, pulmonary tuberculosis, and a smoking history of approximately 1 pack per day.

Upon admission, the patient exhibited tachypnea (RR 35–40/min) and significant ventilator-patient asynchrony. The heart rate was 140 bpm, with a blood pressure of 98/56 mmHg. Bilateral lung auscultation disclosed moist rales, and the patient had cold, clammy skin. Mechanical ventilation were administrated utilizing a pressure control mode with pressure control (PC) set at 19 cmH_2_O, positive end-expiratory pressure (PEEP) at 6 cmH_2_O, and Fraction of inspired oxygen (FiO_2_) of 1. Blood gas analysis revealed a pH of 7.376, Partial pressure of carbon dioxide (PaCO_2)_ of 51 mmHg, partial pressure of oxygen (PaO_2_) of 68.2 mmHg. Laboratory findings showed a white blood cell count of 21.4 × 10^9^/L, C-reactive protein level of 300 mmol/L, procalcitonin level of 13.47 (ng/mL). Patient diagnosis includes: 1. Acute respiratory distress syndrome (ARDS); 2. Septic shock (due to pneumonia and bloodstream infection); 3. Hospital-acquired pneumonia; 4. Hypernatremia; 5. Acute kidney injury (stage III); 6. Esophageal malignancy (post-thoracoscopic esophagectomy for esophageal cancer); 7. Cerebral infarction; Neuromuscular blocking drugs were administered to interrupt the patient’s spontaneous respiratory efforts. Fluid resuscitation and vasopressor support (Norepinephrine, 0.37 μg/kg/min, and Vasopressin at 3 ml/h) were administered to maintain a blood pressure at 130/70 mmHg. The patient received a stable antibiotic regimen based on antimicrobial susceptibility testing, including ceftazidime-avibactam for Klebsiella pneumoniae and sulbactam for Acinetobacter baumannii, with colistin nebulization. After 5 days, improvements in CRP and PCT levels led to discontinuation of intravenous antibiotics by day 7 and nebulization by day 10. Fluid management focused on maintaining blood pressure with a negative balance, except for the initial shock day, and remained relatively stable during NO treatment. A pulse index continuous cardiac output (PICCO) catheter was inserted for hemodynamic monitoring.

**FIGURE 1:**
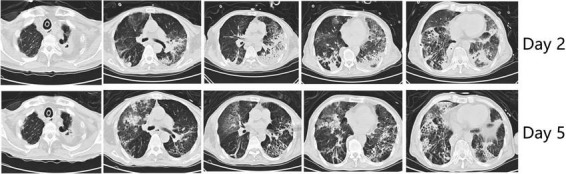
CT images of the lung. Upper of the picture presents CT images of patients at Day 2 and Lower reflects Day 5.

**FIGURE 2:**
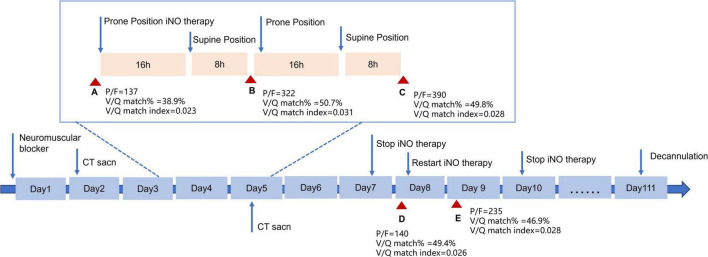
Time line depicting the treatment course of the patients. Red triangle indicates the time detecting ventilation and perfusion by EIT.

On Day 2, a CT scan revealed diffuse bilateral lung infiltration with significant consolidation, particularly in gravity-dependent areas, notably the left lung (Figure 1). Despite stabilizing blood pressure (110/60 mmHg), severe hypoxemia persisted (PaO_2_/FiO_2_ = 77 mm Hg). Therefore, prone positioning for 16 h per day was initiated on Day 3, along with iNO therapy at 15 parts per million (ppm) to enhance oxygenation (Figure 2). At the time of our treatment, no universal guidelines specified a particular iNO dose for ARDS patients. Initial doses typically range from 5–10 ppm, with adjustments based on patient response (4). The maximum iNO dose can reach up to 80 ppm, with no significant toxicity reported (5). In our protocol, we initiated iNO therapy at 10 ppm and increased the dose by 5 ppm every 10 min to monitor SpO_2_ changes. This allowed us to determine the lowest effective dose to maintain SpO_2_ without significant drops (> 10%), ultimately identifying 15 ppm as the optimal dose. To better evaluate lung ventilation and perfusion, EIT examination was employed in real-time during therapy, facilitating the optimization of PEEP titration and determination of treatment timing. Unlike traditional V/Q ratio assessment methods, we introduced cardiac output and minute ventilation to calculate the absolute value of V/Q, and developed a modified index to comprehensively assess the overall pulmonary V/Q situation.

Within 24 h of prone positioning and iNO therapy initiation, the patient’s oxygen index increased from 77 mmHg to 112 mmHg. By Day 5, significant improvement in oxygenation was noted, with the patient maintaining an oxygen index consistently above 200 mmHg during supine positioning. CT scan indicated absorption of bilateral lower lung consolidations. At that time, the patient had evident facial pressure ulcers accompanied by skin bleeding. The patient started to exhibit agitation and intolerance to the prone position despite receiving the standard dosage of sedative medication. Weighing the pros and cons, it was decided to discontinue prone position. On Day 7, iNO therapy was ceased, resulting in a gradual decline in oxygenation index to below 150 mmHg and showed a continuing downward trend. After evaluating the patient’s lung perfusion using EIT, we opted to reintroduce NO inhalation therapy. Consequently, the patient’s oxygenation showed gradual improvement. On Day 10, we ceased the NO inhalation treatment, and the patient maintained satisfactory oxygenation levels. Following anti-infective treatment, sputum clearance, and pulmonary rehabilitation therapies, the patient’s reliance on mechanical ventilation gradually decreased. On the 111th day post-admission, successful extubation was achieved, leading to the patient’s transfer to a convalescent facility for continued care.

### EIT methods and analysis

The EIT belt was placed at the fifth intercostal space around the patient’s chest wall and connected to the EIT monitor (PulmoVista.500; Dräger Medical GmbH, Lübeck, Germany). The patient was deeply sedated and mechanically ventilated on pressure control mode. EIT assessment was conducted with the patient in the supine position before initiating iNO therapy (A), at 24 h after initiation iNO therapy in the prone position (B), at 48 h after initiating iNO therapy in the prone position (C), 24 h after discontinuing iNO therapy (D) and 24 h after restarting iNO therapy (E). To acquire perfusion data, a 10 ml bolus of 10% NaCl solution was injected through the central venous catheter during an 8-s end-expiratory breath hold. Cardiac output was measured using a pulse index continuous cardiac output (PICCO) catheter, and minute ventilation was recorded from the mechanical ventilator simultaneously with EIT measurements. The EIT data were digitally filtered using a low-pass filter with a cut-off frequency of 0.67 Hz to eliminate periodic cardiac-related impedance changes. Offline analysis of the EIT data was performed using software provided by Dräger (Drager EIT Analysis Tool 6.3, 2016), along with algorithms developed by our research team.

Ventilation pixel values were calculated as the impedance change between expiration and inspiration, resulting in the generation of a ventilation map. Pixel values lower than 10% of the maximum value were excluded. Perfusion values of pixels were determined by measuring the slope of the time-impedance curve during the descending phase, providing a relative perfusion image. Pixel values lower than 10% of the maximum value were excluded. To interpret the V/Q ratio obtained through EIT, the following parameters were calculated:

• The Non-CO related parameters: Only perfused fraction is defined as the fraction of perfused pixels relative to the total number of pixels. Only ventilated fraction is defined as the fraction of ventilated pixels relative to the total number of pixels. V/Q match fraction is defined as the both perfused and ventilated pixels divided by the total number of pixels.
• CO-related parameters: The V/Q ratio of each pixel was calculated by dividing absolute ventilation by absolute perfusion, as previously reported (3): V/Q = (V%i × MV × 0.7)/(Q%i × CO). Shunt, low V/Q, normal V/Q, high V/Q and dead space pixels were defined as V/Q ratio ≤ 0.1, 0.1 < V/Q ≤ 0.8, 0.8 < V/Q ≤ 1.25, 1.25 < V/Q ≤ 10 and V/Q ≥ 10, respectively.
  Unlike traditional methods of assessing the V/Q ratio, we incorporated cardiac output and minute ventilation into our calculations to determine the absolute value of V/Q. Additionally, we have developed a modified index that allows for a comprehensive assessment of the overall pulmonary V/Q status.
• CO-related V/Q match index, corresponding to the reciprocal of the variance of the ventilation-perfusion ratio for all pixels relative to 1. For details, CO-related V/Q match index = Var(V/Q)1. Where: Var(V/Q) represents the variance of the ventilation-perfusion ratio (V/Q) for all pixels. V denotes ventilation. Q denotes perfusion. Var indicates variance. The V/Q ratio aligns with the algorithm detailed in the preceding section regarding “CO-related parameters.”

### EIT results

**FIGURE 3:**
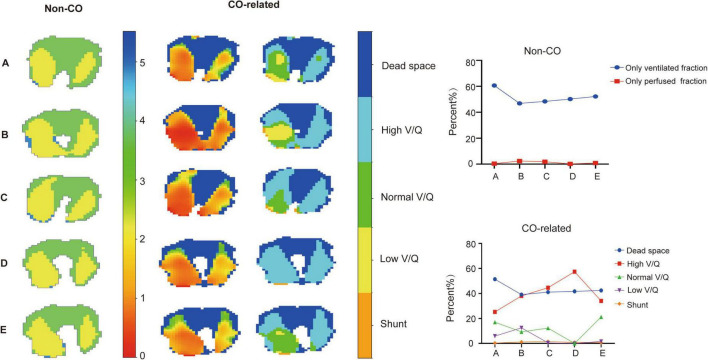
EIT maps of lung V/Q ratio with or without introduction of CO and MV at different time points. The left column shows the overlap of ventilation and perfusion maps using conventional methods. The middle column displays the absolute V/Q ratio heatmap incorporating cardiac output and mechanical ventilation into the analysis. The color gradient ranging from red to blue in the diagram represents the V/Q ratio, which ranges from 0 to 10. The right column exhibits the V/Q ratio map divided into dead space (dark blue), shunt (orange), low V/Q (yellow), normal V/Q (green) and high V/Q (light blue) regions. The line graph shows the change of the specific data of the EIT maps.

In the conventional V/Q map, there was a significant decrease (60.7% vs. 46.9%) in the only perfused regions of the patient after 24 h of iNO therapy, while the only ventilated regions showed minimal changes. In the CO-related V/Q map, the dead space (shunt) regions changed similarly to the only perfused (only ventilated) regions in Non CO-related V/Q maps, with a notable increase in the low V/Q region (5.9% vs. 12.5%) (Figure 3; Table 1). The normal V/Q region of the patient exhibited a downward trend after prone positioning and iNO therapy, experienced a significant decrease after stopping iNO therapy (12.3% vs. 0%) (Table 1), and quickly rebounded upon resumption of iNO therapy (0% vs. 21.2%) (Table 1), while the change trend of the high V/Q region was the opposite (Figure 3).

**TABLE 1:** The EIT derived parameters of patients in different time points.

	A	B	C	D	E
Time point	Before iNO therapy and prone position	24 h after iNO	48 h after iNO	After stopping iNO	24 h after restarting iNO therapy
Dead space (%)	60.7	46.9	48.4	50.2	52.2
Shunt (%)	0.4	2.4	1.9	0.23	0.88
VQ match (%)	38.9	50.7	49.8	49.4	46.9
Dead space (%)	51.4	39.1	41.1	41.7	42.4
High V/Q (%)	25.2	38.0	44.6	57.4	34.0
Normal V/Q (%)	17.0	9.3	12.3	0	21.2
Low V/Q (%)	5.9	12.5	0.5	0	1.6
Shunt (%)	0.34	1.2	1.5	0.89	0.72
V/Q match index	0.023	0.031	0.028	0.026	0.028
**Artery blood gas**					
P/F (mmHg)	77	112	390	140	235
PaCO_2_ (mmHg)	37.7	43.8	54.7	56.9	53.3
**Ventilator parameters**					
PC (cmH_2_O)	21	21	20	20	19
PEEP (cmH_2_O)	8	8	6	6	6
P_peak_	30	30	27	26	25
Pplat	19	19	16	16	15
FiO_2_ (%)	70	50	50	70	60
Vt (mL)	348	373	430	406	371
MV (L/min)	10.6	11	13	12.8	10.9
Cardiac output (L/min)	5	5.1	4.3	3.1	5

**FIGURE 4:**
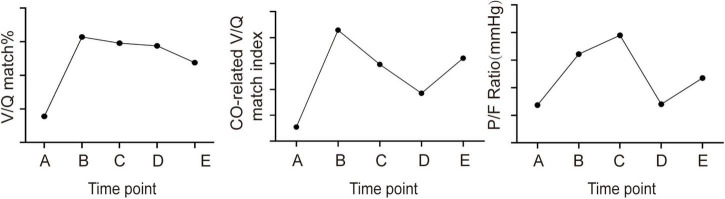
The change trend of V/Q match fraction, CO-related V/Q match index, and patient oxygenation index at different time points.

After 24 h of iNO inhalation therapy, V/Q matching regions significantly increased (50.7% vs. 38.9%) (Figure 4; Table 1), and then gradually declined. The CO-related V/Q match index also increased after the initial 24 h (0.031 vs. 0.028) (Table 1) and decreased after iNO therapy cessation (0.026 vs. 0.028) (Table 1), but rebounded after resuming iNO therapy (0.028 vs. 0.026) (Table 1), aligning with the trend in the patient’s oxygenation index (Figure 4).

## Discussion

This case presents a biologic model to explore the capability of a modified EIT-derived parameter, CO-related V/Q match index, in assessing V/Q ratio in patients undergoing prone position and iNO therapy. It also revealed, for the first time, the noticeable increase in the absolute V/Q ratio, as indicated by the CO-related V/Q match index, resulting from the implementation of iNO.

Since 1993, iNO has been extensively studied as a rescue therapy for patients with ARDS (4). However, the overall mortality of ARDS patients did not show significant differences after iNO therapy (6). One possible explanation is the challenge in identifying which patients may benefit the most from iNO therapy (4). iNO works by selectively dilating pulmonary blood vessels, improving V/Q matching by increasing perfusion in well-ventilated areas of the lung (7). In this case, the patient had a high proportion of dead space, suggesting a potential benefit from iNO compared to those with a high proportion of shunt. While it may have been difficult to attribute the improvement in oxygenation solely to iNO, especially in the presence of prone positioning during early treatment, the subsequent decline in oxygenation and decrease in the V/Q ratio upon discontinuing iNO, followed by improvement after resuming iNO therapy, suggests the beneficial impact of iNO in the patient’s treatment. Besides, during the NO inhalation period, the patient’s antibiotic regimen, fluid management, and mechanical ventilation settings remained relatively stable, which allows us to partially exclude these factors as significant confounders.

EIT has emerged as a powerful tool for monitoring ventilation-perfusion (V/Q) matching in ARDS, offering real-time insights that are crucial for guiding therapeutic interventions such as PEEP optimization (8), prone positioning, and inhaled nitric oxide (iNO) therapy (9, 10). In traditional V/Q assessment, the V/Q match fraction was calculated by combining ventilation and perfusion maps to determine the proportion of lung areas that were both ventilated and perfused (11). However, this approach lacked explicit numerical values for the V/Q ratio, which omitted important information about lung V/Q match. Recent studies have highlighted the critical role of incorporating cardiac output (CO) in EIT-based V/Q assessments, demonstrating that neglecting CO can introduce significant bias, particularly in patients with an alveolar ventilation to cardiac output (VA/QC) ratio greater than 1 (3). This finding emphasizes the need for accurate calibration methods to ensure reliable V/Q assessments. Our study introduces a novel CO-related V/Q match index, which builds on these insights by providing a more precise measure of V/Q matching. In this particular case, the patient experienced three fluctuations during treatment, but the V/Q match fraction only showed a significant increase during the first fluctuation (Figure 4). The failure of the V/Q match fraction to reflect changes in the patient’s condition during the second and third fluctuations can be attributed to the fact that these changes were primarily driven by alterations in ventilation and perfusion proportions, rather than the fraction of areas with both ventilation and perfusion. This assumption is supported by the use of absolute V/Q ratio measurements. During the second fluctuation, the patient’s MV remained relatively stable (12.8 L vs. 13 L) while the CO significantly decreased (3.1 L vs. 4.3 L) (Table 1). As a result, the overall absolute V/Q ratio increased, leading to a reduction in areas with a normal V/Q ratio and an increase in areas with a high V/Q ratio (Figure 3). However, the different classifications of V/Q regions only show changes in their respective proportions. A single value change cannot represent the overall V/Q match of the lung, and therefore, none of these parameters correlates well with the patient’s oxygenation index. Our parameter combines the advantages of the above two methods while addressing their limitations. It calculates the absolute value of V/Q for each pixel of lung using CO and MV, thereby maximizing the information content and accuracy. It reflects the V/Q match of the whole lung region in the way of variance, providing a simple and intuitive quantitative index for the overall match of lung ventilation and perfusion.

Currently, CO can be measured using both invasive and non-invasive methods. Invasive techniques provide accurate but require arterial catheterization and carry potential risks. Non-invasive methods, such as transthoracic echocardiography (TTE) and the Ultrasound Cardiac Output Monitor (USCOM) (12), offer comparable accuracy to invasive techniques and are more feasible for use in the ICU setting. Electrical impedance tomography (EIT) offers a non-invasive alternative for CO monitoring by analyzing thoracic electrical bioimpedance (13). Recent studies have shown that EIT can be calibrated to provide CO measurements, even without invasive monitoring, by using the pulsatility signal in the EIT data (14). This approach has been validated in ARDS patients, demonstrating good agreement with invasive methods. EIT-derived CO can also be used to assess absolute V/Q, which enhances the clinical utility of EIT in monitoring and guiding treatments for ARDS (14). This advancement allows for the application of these methods in calculating our novel V/Q parameter, thereby broadening its applicability.

The limitations of this case include the restricted number of EIT assessment time points, which hindered a robust validation of the modified parameter’s efficacy. Additionally, while the optimal V/Q ratio can span a range from 0.8 to 1.0, our analysis exclusively selected 1.0 as the ideal value for calculating the modified index relative to this benchmark. This approach may have partially influenced the outcomes, particularly for patients whose predominant V/Q ratio was 0.8. Despite the relative stability of our treatment protocols during the NO inhalation period, our case did not explore the relationship between other therapies and the P/F ratio, such as antibiotic therapy or fluid balance. Additionally, integrating the CO-related V/Q match index into routine clinical practice faces challenges, including the lack of standardization in lung perfusion assessment using EIT and the complexity added by the invasive nature of the PiCCO method for measuring cardiac output. The generalizability of our findings is limited due to the single-case nature of the study, and further validation in larger, diverse cohorts is needed.

## Conclusion

In conclusion, this case elucidates the significance of the modified EIT-derived parameter——CO-related V/Q match index, revealing its benefits in evaluating the V/Q ratio under the various treatment strategies when compared to traditional ones.

## Data Availability

The original contributions presented in this study are included in this article/supplementary material, further inquiries can be directed to the corresponding authors.
